# Weak Expression of Terminal Complement in Active Antibody-Mediated Rejection of the Kidney

**DOI:** 10.3389/fimmu.2022.845301

**Published:** 2022-04-13

**Authors:** Gesa Tiller, Rosa G. M. Lammerts, Jessy J. Karijosemito, Firas F. Alkaff, Arjan Diepstra, Robert A. Pol, Anita H. Meter-Arkema, Marc. A. Seelen, Marius C. van den Heuvel, Bouke G. Hepkema, Mohamed R. Daha, Jacob van den Born, Stefan P. Berger

**Affiliations:** ^1^ Division of Nephrology, Department of Internal Medicine, University Medical Center Groningen, Groningen, Netherlands; ^2^ Department of Laboratory Medicine, University Medical Center Groningen, University of Groningen, Groningen, Netherlands; ^3^ Division of Pharmacology and Therapy, Department of Anatomy, Histology, and Pharmacology, Faculty of Medicine Universitas Airlangga, Surabaya, Indonesia; ^4^ Division of Pathology, Department of Pathology and Medical Biology, University of Groningen, University Medical Center Groningen, Groningen, Netherlands; ^5^ Department of Surgery, University Medical Center Groningen, University of Groningen, Groningen, Netherlands; ^6^ Department of Nephrology, University of Leiden, Leiden, Netherlands

**Keywords:** antibody-mediated rejection, kidney transplantation, complement system, membrane-attack complex, C5b-9

## Abstract

**Background:**

The role of the complement system in antibody-mediated rejection (ABMR) is insufficiently understood. We aimed to investigate the role of local and systemic complement activation in active (aABMR). We quantified complement activation markers, C3, C3d, and C5b-9 in plasma of aABMR, and acute T-cell mediated rejection (aTCMR), and non-rejection kidney transplant recipients. Intra-renal complement markers were analyzed as C4d, C3d, C5b-9, and CD59 deposition. We examined *in vitro* complement activation and CD59 expression on renal endothelial cells upon incubation with human leukocyte antigen antibodies.

**Methods:**

We included 50 kidney transplant recipients, who we histopathologically classified as aABMR (n=17), aTCMR (n=18), and non-rejection patients (n=15).

**Results:**

Complement activation in plasma did not differ across groups. C3d and C4d deposition were discriminative for aABMR diagnosis. Particularly, C3d deposition was stronger in glomerular (P<0,01), and peritubular capillaries (P<0,05) comparing aABMR to aTCMR rejection and non-rejection biopsies. In contrast to C3d, C5b-9 was only mildly expressed across all groups. For C5b-9, no significant difference between aABMR and non-rejection biopsies regarding peritubular and glomerular C5b-9 deposition was evident. We replicated these findings *in vitro* using renal endothelial cells and found complement pathway activation with C4d and C3d, but without terminal C5b-9 deposition. Complement regulator CD59 was variably present in biopsies and constitutively expressed on renal endothelial cells *in vitro*.

**Conclusion:**

Our results indicate that terminal complement might only play a minor role in late aABMR, possibly indicating the need to re-evaluate the applicability of terminal complement inhibitors as treatment for aABMR.

## Introduction

Antibody-mediated rejection (ABMR) is a major reason for impaired function and reduced longevity of the kidney allograft, accounting for about 60% of chronic graft failure in kidney transplant recipients (KTR) (1–5). Understanding the underlying pathoimmunological processes associated with ABMR is crucial for accurate diagnosis and implementation of effective treatment strategies. Former Banff classifications identified ABMR based on the presence of donor-specific HLA-antibodies (DSA), C4d complement deposition, microvascular inflammation, and/or transplant glomerulopathy in renal specimens (6).

From a traditional perspective, ABMR pathomechanism was thought to be based on the activation of the classical complement pathway, initiated by HLA-Antibodies (HLA-Abs), subsequent binding of C1q, C3-convertase formation, and final cellular cytotoxic damage through C5b-9 (2, 7–11). C4d, a split product of the classical and lectin pathway, is relatively stable and binds covalently to renal capillaries. Therefore, C4d was introduced and is still utilized as a diagnostic marker for complement pathway activation (8, 12).

However, recent Banff classifications recognize ABMR subtypes, including C4d-negative ABMR and C4d-positive ABMR without DSA, thereby questioning the universal pathogenic role of the classical complement pathway in ABMR (4, 13–15). These Banff updates indicate that ABMR encompasses a more heterogeneous group, which is possibly linked to diverse pathomechanisms. Moreover, studies evaluating the efficacy of terminal complement inhibitors as a potential treatment for ABMR, showed that terminal complement inhibition was insufficient in preventing ABMR occurrence in the long term (13, 16, 17).

A possible reason for the lack of C4d deposition in the biopsies of some ABMR patients and the insufficient efficacy of complement inhibitors might be that the classical complement pathway does not serve as a universal pathogenetic model for ABMR. Coherently, a recent study by Mező et al. (2019) showed that complement factors in plasma failed to predict ABMR outcome (18).

A more nuanced view on the role of the complement system in ABMR is necessary to precisely define the role of the complement system in its development. However, evaluation of the full complement orchestra with analysis of the consecutive steps of the classical complement pathway activation in ABMR is still missing. We aimed to analyze the role of the classical complement pathway in ABMR, specifically active ABMR, by quantification of representative complement activation products, both in plasma, in kidney transplant biopsies, and upon HLA-Ab binding to renal endothelial cells *in vitro.*

## Materials and Methods

### Study Population and Sample Collection

The study population encompassed a total of 227 KTR of whom blood samples for direct complement measurement were taken and who had either an indication or protocol kidney biopsy at the University Medical Center Groningen (UMCG), the Netherlands, between 2010 and 2020. KTR were recruited with informed consent in the *Transplantlines biobank cohort* study, following the Declarations of Helsinki and Istanbul, with NCT02811835 as ClinicalTrials.gov identifier. The research was approved by the UMCG institutional review board (METc 2008/186) (19).

**Figure 1:**
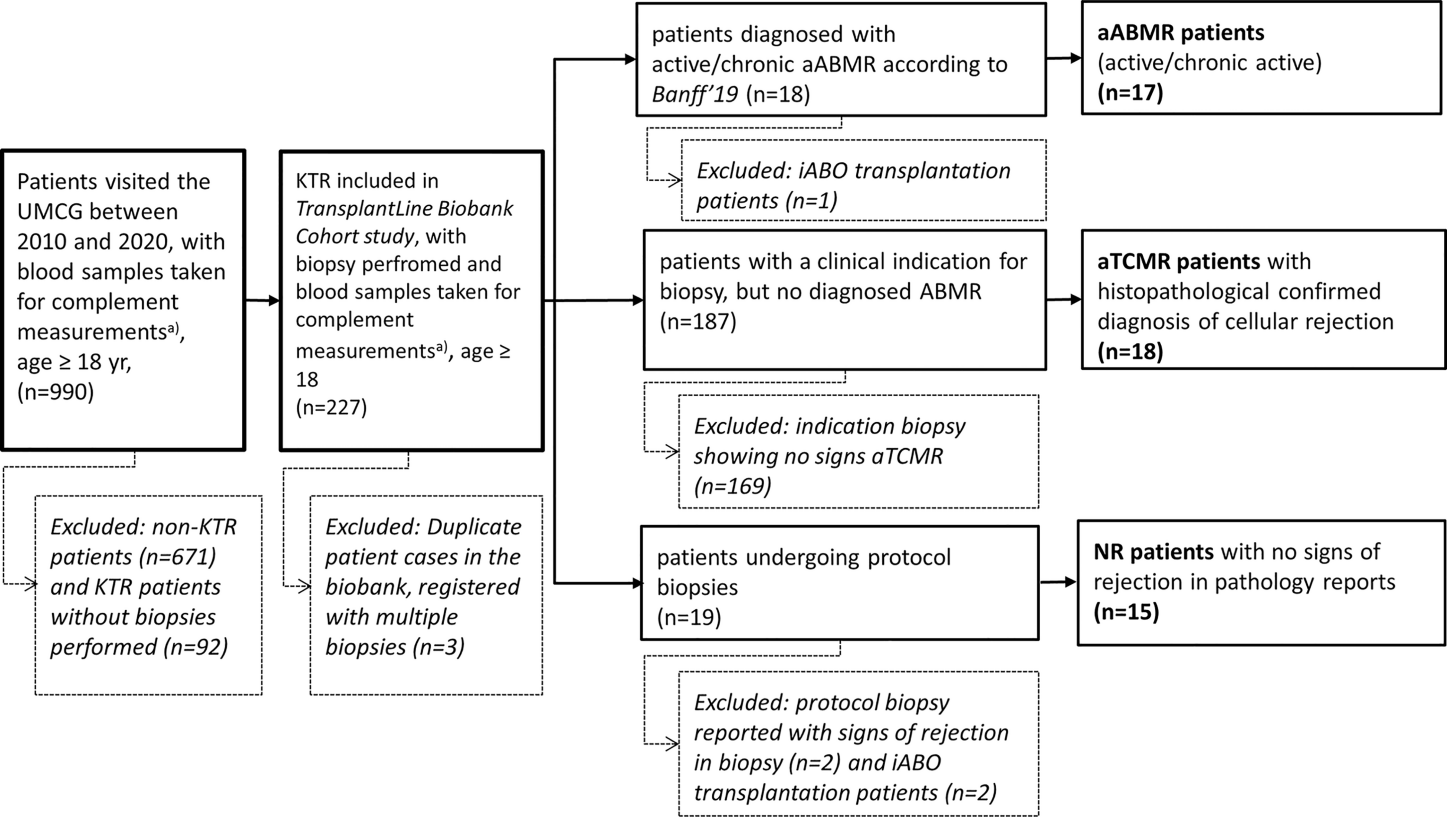
Patient Selection with Inclusion and Exclusion Criteria. Flow chart representing selection of patients based on exclusion criteria. Total amount of patients excluded and included are depicted as (n). Exclusion of patients is presented in box with dotted lines. KTR, Kidney Transplant Recipients; UMCG, University Medical Center; yr, years; non-KTR, patients without a renal transplant; aABMR, active antibody-mediated rejection; iABO, blood group incompatible; aTCMR, acute T-cell mediated rejection; NR, non-rejection. a) with blood samples directly placed on ice.

KTRs were retrospectively included and classified as aABMR, acute T-cell mediated rejection (aTCMR), and non-rejection (NR) patients. All NR patients had a protocol biopsy taken without signs of rejection. Inclusion and exclusion criteria are depicted in **Figure 1**. 50 KTR were included, of whom 17 were classified as aABMR, 18 as aTCMR patients, and 15 as NR patients. As included patients were diagnosed based on different versions of the Banff guideline depending on the year of consultation, standardized re-evaluation of the diagnosis by the most recent Banff’19 guideline was performed. Re-evaluation according to Banff’19 classification considered presence of HLA-Abs, signs of microvascular inflammation in the biopsy, and C4d deposition (15). In case antibody screening in serum revealed no HLA-Abs in pre-categorized cases of aABMR, we only included these HLA-Abs-negative patients as aABMR patients, if otherwise clear histopathological signs of microvascular inflammation were present. Coherently, C4d-negative patients were only included as aABMR patients, if histopathological microvascular inflammation was unambiguously identified and serum samples were positive for either HLA-Abs class I or II or both (15).

Plasma samples taken closest to the date of biopsy were immediately stored at minus 80°C and only thawn directly prior to complement measurements. Clinical data regarding patient demographics, underlying renal disease, transplantation, immunological status, and kidney function around the time of sampling were derived from the hospital electronic patient file (Epic Systems Cooperation, EPIC, Wisconsin, United States). Kidney function was assessed by estimating glomerular filtration rate (eGFR) by applying the Chronic Kidney Disease Epidemiology Collaboration equation (20). Protein excretion of ≥ 0.5 g per day was defined as proteinuria. Renal biopsy samples were taken based on clinical indication or in the context of protocol biopsies, fixed in formalin, embedded, and stored in paraffin.

### Human-Leucocyte Antibody Diagnostics and Complement Quantifications

#### Detection of Donor-Specific Antibodies

The presence of HLA-Abs class I and II n serum was evaluated prior to anti-rejection therapy using the Life screen the Luxe (LsdL), in accordance with the manufacturer’s protocol (Immucor Transplant Diagnostics, Norcross, Georgia). Serum samples of aABMR and aTCMR patients, which were tested positive in LsdL class I and/or II were subsequently tested with Lifecodes Single Antigen Bead (LSA;Immucor Transplant Diagnostics) class I and/or II assay respectively. In light of the HLA-donor type, LSA test results were evaluated regarding donor-specificity and mean fluorescence intensity (MFI) by B.G.H. and R.G.L. (Department of Transplant Immunology, Groningen).

#### Quantification of Systemic C3, C3d, and C5b-9

C3 quantification in patients’ plasma was performed using radial immunodiffusion assay (RID) measurement according to Mancini et al. (21). C3d and soluble C5b-9 were quantified by enzyme-linked immunosorbent assay (ELISA) (22, 23). C3d measurements were preceded by Polyethylene Glycol precipitation for fractionation of C3d (22). For accurate quantification, standard curves were derived from a serial dilution with a standard sample kindly provided by the University of Leiden for C3d; and by serial dilution of zymosan-activated normal human serum in PTB [PBS (Phosphate Buffered Saline) with 0.05% Tween and 1% Bovin Serum Albumin (BSA)] of defined concentration for C5b-9. The C3d/C3 ratio was determined by dividing the C3d values in ng/mL by the C3 concentration in ng/mL. Details on antibodies and conjugates used for quantification of respective complement factors are provided in **Table 1**.

**Table 1:** Antibodies for ELISA on plasma samples.

**Target**	**Primary antibody**	**Secondary antibody**		**Tertiary antibody**	**Substrate**	**Stop solution**	**Sample diluent (solution)**
**C5b-9**	Monoclonal mouse anti-human C5b-9, (Dako, MO777) (1:1000)	Polyclonal goat anti-human C5, (Quidel, Ca92121) (1:1000)	Polyclonal mouse anti-goat HRP, (Jackson, 205-035-108) (1:5000)		TMB (Sigma, T0440)	1M H2S04	PTB (PBS with 0.05% Tween, 1% BSA) and 0,01M EDTA (1:3)
**C3d**	Polyclonal rabbit anti-human C3d, (Dako, A0063) (1:1000)	Polyclonal anti-human C3d-DIG, (Dako) (1:10000)	Polyclonal anti-DIG, (Roche, 11207733910) (1:8000)		ABTS (Sigma, A1888)	N/A	PTB (PBS with 0.05% Tween, 1% BSA) and 0,01M EDTA (1:100)

Antibodies for ELISA analysis of plasma samples from ABMR, aTCMR, and NR patients. Applied dilutions are given below each antibody used. TMB, 3,3’,5,5’-tetramethylbenzidine; N/A, not applicable; ABTS, 2,2′-Azino-bis(3-ethylbenzthiazoline-6-sulfonic acid); PBS, Phosphate Buffered Saline.

### Staining of C4d, C3d, C5b-9 and CD59 in Renal Biopsies

Paraffin-embedded renal specimens were stained with rabbit anti-human C4d (clone SP91, Ventana, Benchmark automated immunostainer) according to the manufacturer’s protocol, as part of the standardized pathological evaluation of biopsies. C3d, C5b-9, and CD59 staining were performed using in-house stainings. Details on C4d, C3d, C5b-9, and CD59 staining in renal biopsies are given in **Table 2**. Solutions were prepared with BSA (A9647, Sigma, St Louis, United States) and PBS (17-512Q, Lonza, Wijchen, The Netherlands) with specific dilutions provided in **Table 2**. Between every incubation step, slides were washed three times with PBS (17-512Q, Lonza, Wijchen, The Netherlands).

**Table 2:** Complement factor staining in biopsies from kidney transplant recipients.

Antigen	Fixation and Embedment	Deparaffinization	Antigen retrieval	Blocking steps	Antibody	Conjugate 1	Conjugate 2	Substrate	Counterstaining and embedment	Control sample
**C3d**	Paraffin-embedded tissue	Xylol and Alcohol (100%,96%, 70%) and Demi-water in 6 consecutive steps	Incubation in dark for 30 min, 37°C with 0,4% pepsin from porcine stomach mucosa in 37% 0,1N HCl in demi-water (pH=2.5)	First, 0,01% H2O2 (in 1% BSA with PBS) Second, blocking step with 1% BSA in PBS	Polyclonal rabbit anti-human C3d (A0063, Dako) at 4°C overnight 1:2500 dilution in 1% BSA in PBS	HRP on polyclonal goat anti-rabbit antibody (Dako, P0448) at room temperature 1:100 in 1% BSA in PBS	HRP on polyclonal rabbit anti-goat antibody (Dako, P0449) at room temperature 1:100 dilution in 1% BSA in PBS	0.2 mg/ml 3-Amino-9-ethylcarbazole (Sigma 02431MH), in 50mM Acetate buffer and 0.03%H2O2, (pH=5.5)	Hematoxylin 1:2 for 5 seconds Kaiser’s glycerol gelatine embedment (1092420100, Merck, Darmstadt, Germany)	Kidney biopsy of a patient with systemic lupus erythematosus; Pre-transplantation biopsy of non-heart-beating and living donor
**C5b-9**	Paraffin-embedded tissue	Xylol and Alcohol (100%,96%, 70%) and Demi-water in 6 consecutive steps	Incubation for 30 min in dark at room temperature with 0,1% protease	0,01% H202 in PBS	Monoclonal mouse anti-human C5b-9 (Quidel, A239) at 4°C overnight 1:10000 dilution in 1% BSA in PBS	HRP on polyclonal rabbit anti-mouse antibody (Dako, P0260) at room temperature 1:100 dilution in 1% BSA in PBS+1%NHS	HRP on polyclonal goat anti-rabbit antibody (Dako, P0448) at room temperature 1:100 dilution in 1% BSA in PBS+1%NHS	0.2 mg/ml 3-Amino-9-ethylcarbazole (Sigma 02431MH) in 50mM and 0.03%H2O2, (pH=5.5)	Hematoxylin 1:2 for 5 seconds Kaiser’s glycerol gelatine embedment (1092420100, Merck, Darmstadt, Germany)	Pre-transplantation biopsy of non-heart beating donor
**CD59**	Paraffin-embedded tissue	Xylol and Alcohol (100%,96%, 70%) and Demi-water in 6 consecutive steps	Heat-induced epitope retrieval with Pascal pressure-cooker (DakoCytomation, Glostrup, Denmark) at 115°C for 7 min in 0.2N HCl in Demi-water	0,3% H202 in PBS	Monoclonal mouse anti-human CD59 (Hycult, HM2120) at room temperature for 60 minutes 1:50 dilution in 1% BSA in PBS	HRP on polyclonal rabbit anti-mouse antibody (Dako, P0260) at room temperature 1:100 dilution in 1% BSA in PBS	HRP on polyclonal goat anti-rabbit antibody (Dako, P0448) at room temperature 1:10 dilution in 1% BSA in PBS	0.5 mg/ml 3,3’-Diaminobenzidine (Merck, D5637) incubation for 20 min, in dark at room temperature in PBS + 0,3% H202	Periodic Acid-Schiff counterstaining for 5 and 15 min respectively embedment in Permount™ Mounting Medium (FisherScientific, Loughborough, United Kingdom) after dehydration	Pre-transplantation biopsy of non-heart beating and living donor kidney

HCl, Hydrochloric acid; BSA, bovine serum albumin; HRP, Horseradish peroxidase; NHS, N-Hydroxysuccinimide; PBS, Phosphate Buffered Solution.

A renal pathologist (M. vd H.) blindly scored the presence of peritubular capillary C4d according to the recent Banff`19 classification standard (15). For inter-group statistical analysis, semiquantitative scores of the pathologist were categorized as ‘C4d positive’ or ‘C4d negative’.

Slides stained for C3d and C5b-9 were digitalized by the Department of Pathology & Medical Biology of the UMCG, using a Hamamatsu slide scanner (Hamamatsu Photonics, Hamamatsu, Japan). Stained slides were blindly graded by M. vd H. in the Aperio ImageScope software (Leica Biosystems, Nussloch, Germany). Complement factors C3d and C5b-9 were scored with a semiquantitative scoring system previously described by Bobka et al. (2018) as a method of analyzing complement deposition in biopsies (24). Glomerular and peritubular semiquantitative scores ranged from 0 to 4, indicating lack of any deposit, weak (≤25%), moderate (≤50%), substantial (≤75%), and intense deposition.

### Antibody-Mediated Complement Factor Activations in a Renal Endothelial Cell Model

To evaluate whether HLA-Abs can activate the complement cascade up to C5b-9 on renal endothelial cells, *in vitro* experiments using conditionally immortalized glomerular endothelial cells (CiGEnCs) were conducted (25). In addition, the presence of terminal complement regulator CD59 was analyzed. Details on handling and characterization of the CiGenCs cell-line are provided in the **Supplementary Material 2A**. Depending on the incubation condition of the complement activation assay, cells were incubated with either human monoclonal HLA-antibodies (A2/A28 IgG1, provided by Dr. F. Claas, Leiden University Medical Center, The Netherlands), or 25% human serum containing HLA-A2 antibodies directed against HLA antigens expressed on the CiGEnC cell membrane, or a pan-HLA-class I antibody (clone W6/32). 25% heat-inactivated blood group compatible serum was used as a control. The following washing steps are described in **Supplementary Material 2B**. For detection of activated C3, C4d, C5b-9, and CD59, cells were incubated with primary and secondary antibodies, as depicted in **Table 3**. To exclude apoptotic and necrotic cells, propidium iodide 1 μg/ml (Molecular Probes, Leiden, The Netherlands) was added just before measuring. Activated C3, C4d, and neoantigen C9 deposition on viable non-apoptotic cells were analyzed in a FACSCalibur™ (FACSCalibur, Becton Dickinson, New Jersey, USA). Results were derived from three independent experiments.

**Table 3:** Antibodies for flow cytometric analysis on CiGEnCs.

Target	Activated C3	C4d	C5b-9	CD59
**Primary antibody**	Monoclonal Mouse anti-human activated C3 antibody, which recognizes C3b, iC3b, and C3c fragments (Clone bH6, HM2168S, Hycult biotech, Uden, The Netherlands) 1:50	Mouse anti-human C4d antibody (Clone 12D11, HM2229 20UG, Hycult biotech, Uden, The Netherlands) 1:100	Mouse anti-human neoantigen-C9 antibody (HM2264, Hycult biotech, Uden, The Netherlands) 1:100	Mouse anti-human CD59 (HM2120, Hycult biotech, Uden, The Netherlands) 1:100
**Secondary antibody**	Goat anti-mouse FITC-labelled antibody (Cat. No. 1031-02, SouthernBioTech, Uden, The Netherlands) 1:100	Goat anti-mouse FITC-labelled antibody (Cat. No. 1031-02, SouthernBioTech, Uden, The Netherlands) 1:100	Goat anti-mouse FITC-labelled antibody (Cat. No. 1031-02, SouthernBioTech, Uden, The Netherlands) 1:100	Goat anti-mouse FITC-labelled antibody (Cat. No. 1031-02, SouthernBioTech, Uden, The Netherlands) 1:100

Incubation with primary antibodies was performed on ice for 30 min. Incubation with secondary antibodies was conducted in dark for an additional time of 30 min.

### Statistics

Statistical tests were conducted for the baseline characteristics and complement component measurements. Normality was examined by the Kolmogorov Smirnov test for each variable. Categorical parameters are represented as n (%). For continuous data, normally distributed variables are expressed as mean ± standard deviation (SD) and skewed data as median and interquartile range (IQR). All three groups were analyzed by Kruskal-Wallis for non-parametric variables and by one-way ANOVA for parametric variables. Analyses between subgroups were additionally performed for complement measures in serum and biopsy, comparing aABMR with aTCMR patients, and aABMR with NR patients. These two-group analyses were conducted by Mann-Whitney U test for non-parametric and by independent unpaired t-test for parametric data. Correlations between plasma and local complement factors were derived from Spearman’s correlation analyses. Computations were performed by SPSS (IBM SPSS Statistics, Chicago, United States) version 23, and GraphPad software 8.0 (GraphPad Software, San Diego, United States) was used for graphical visualization.

## Results

### Study Population

Among 50 KTR, 17 were identified as aABMR patients according to the Banff ‘19 classification, 18 as aTCMR, and 15 as NR (**Figure 1** **)** (2, 4). Mean age at biopsy was 46.8 ± 12.5 years in the ABMR group, 48.1 ± 16.8 years in the aTCMR group, and 51.1 ± 15.3 years in the NR group. No significant differences were found in demographic characteristics between all groups (**Table 4**). We included ABMR patients based on biopsy specimens with clear histopathological evidence for ongoing rejection, and only considered cases of active or chronic active ABMR, following the Banff’19 criteria (15). Thus, KTR with chronic, inactive ABMR were excluded (**Figure 1** and **Table 5**). Within the aABMR group, 3 patients showed acute active ABMR, with the diagnosis made within the first 30 days after transplantation (**Table 5** **).** Based on inclusion criteria, all transplants were ABO-compatible.

**Table 4:** Baseline characteristics of aABMR, aTCMR, and NR patients.

Variable	aABMR* *n = 17*	aTCMR* *n = 18*	NR *n = 15*	*p*
**Recipient**				
Age at time of transplantation in (yr, mean ± SD)	43,9 ± 13,9	47,5 ± 17,7	49,9 ± 15,6	0,47
Type of transplantation				**0,04**
Living Related (n, %)	3 (17,6)	3 (16,7)	6 (40)	
Living Unrelated (n, %)	7 (41,2)	2 (11,1)	5 (33,3)	
Donation after Brain Death (n, %)	6 (35,5)	9 (50)	3 (20)	
Donation after Cardiac Death (n, %)	1 (5,9)	4 (22,2)	1 (6,7)	
Gender (n, %)				0,07
Female sex	4 (23,5)	11 (61,1)	5 (33,3)	
Primary renal disease (n, %)				0,73
Diabetic nephropathy	0 (0)	2 (11,1)	0 (0)	
IgA nephropathy	4 (23,5)	1 (5,6)	5 (33,3)	
Glomerulonephritis	0 (0)	2 (11,1)	0 (0)	
Polycystic kidney disease	3 (17,6)	5 (27,8)	1 (6,7)	
Pyelonephritis	0 (0)	0 (0)	0 (0)	
Hypertensive nephropathy	3 (17,6)	3 (16,7)	1 (6,7)	
MPO-vasculitis	0 (0)	0 (0)	2 (13,3)	
Unknown	2 (11,8)	0 (0)	3 (20)	
Other	5 (29,4)	5 (27,8)	3 (20)	
**Donor**				
Gender (n, %)				0,68
Female sex	6 (35,3)	7 (38,9)	8 (53,3)	
missing	4 (23,5)	1 (5,6)	1 (6,7)	
Age at time of donation (yr, median ± IQR)	47 ± 20	54 ± 13)	54,5 ± 20	0,09
**Transplant and Transplantation characteristics**				
HLA mismatches (n, %)				
mismatches of HLA-AB				0,13
0	0 (0)	4 (22,2)	1 (6,7)	
1	2 (11,8)	4 (21,1)	2 (13,3)	
2	9 (52,9)	6 (33,3)	7 (46,7)	
3	4 (23,5)	2 (11,1)	2 (13,3)	
4	1 (5,9)	1 (5,6)	1 (6,7)	
missing	1 (5,9)	1 (5,6)	2 (13,3)	
mismatches of HLA- DR				0,32
0	4 (23,5)	6 (33,3)	3 (20)	
1	7 (41,2)	10 (55,6)	6 (40)	
2	4 (23,5)	1 (5,6)	4 (26,7)	
missing	2 (11,8)	1 (5,6)	2 (13,3)	
CMV serological status (n, %)				0,08
Donor-/recipient-	7 (41,2)	1 (5,6)	2 (13,3)	
Donor+/recipient-	5 (29,2)	6 (33,3)	4 (26,7)	
Donor-/recipient+	1 (5,9)	5 (27,3)	3 (20)	
Donor+/recipient+	4 (23,5)	6 (33,3)	6 (40)	
**Biopsy**				
Time since transplantation (yr, mean ± std)	4,4 ± 3,4	1 ± 1,4	0,8 ± 0,3	**<0,01**
Age at biopsy (yr, mean ± SD)	46,8 ± 12,5	48,1 ± 16,8	51,1 ± 15,3	0,71
Rationale for undergoing biopsy (n, %)				**<0,01**
Delayed graft function	1 (5,9)	4 (22,2)	0 (0)	
elevated creatinine level	4 (23,5)	4 (22,2)	0 (0)	
proteinuria (n, %)	8 (47,1)	1 (5,6)	0 (0)	
elevated creatinine and proteinuria	0 (0)	3 (16,7)	0 (0)	
cyst formation	0 (0)	1 (5,6)	0 (0)	
BK viremia	0 (0)	1 (5,6)	0 (0)	
transplantectomy	2 (11,8)	0 (0)	0 (0)	
general kidney function decline	2 (11,8)	4 (22,2)	0 (0)	
protocol	0 (0)	0 (0)	15 (100)	
C4d in biopsy (n, %)				**<0,01**
positive	16 (94,1)	1 (5,6)	0 (0)	
negative	1 (5,9)	17 (94,4)	15 (100)	
SV40 in biopsy (n, %)				0,32
positive	0 (0)	1 (5,6)	1 (6,7)	
negative	15 (88,2)	17 (94,4)	13 (86,7)	
missing	2 (11,8)	0 (0)	1 (6,7)	
**Laboratory measurements**				
LsdL class I (n, %)				**<0,01**
positive	11 (64,7)	1 (5,6)	0 (0)	
negative	6 (35,5)	13 (72,2)	3 (20)	
missing	0 (0)	4 (22,2)	12 (80)	
LsdL class II positive (n, %)				**<0,01**
positive	14 (82,4)	3 (16,7)	0 (0)	
negative	3 (17,6)	11 (61,1)	3 (20)	
missing	0 (0)	4 (22,2)	12 (80)	
LsdL class I and I (n, %)				**<0,01**
LsdL class I and II both negative	1 (5,9)	11 (61,1)	3 (20)	
At least one of LsdL class I or class II positive	16 (94,1)	3 (16,7)	0 (0)	
missing	0 (0)	4 (22,2)	12 (80)	
eGFR (ml/min/1.73m, median ± IQR)	26 ± 19	17,5 ± 12,1	49 ± 18	**<0,01**
Creatinine Clearance (ml/min, median ± IQR)	34 ± 37	23 ± 15	70 ± 21	**<0,01**
Protein in 24-hour Urine Samples (g/24 h, median ± IQR)	1,5 ± 1,9	0,3 ± 1,0	0,2 ± 0,1	**<0,01**
Proteinuria (>0.5g/24h, n, %)				**<0,01**
yes	13 (76,5)	7 (38,9)	0 (0)	
no	1 (5,9)	10 (55,6)	12 (80)	
missing	3 (17,6)	1 (5,6)	3 (20)	

Categorical and dichotomous variables are presented as absolute numbers and respective percentages. Continuous, non-parametric variables are presented as median and with interquartile range. Continuous, parametric data is provided as mean and standard deviation. Categorical data, as well as parametric continuous variables, are compared between groups by ANOVA, continuous non-parametric by Kruskal-Wallis test. Statistical significance is defined as P < 0.05 (bold). aABMR, active antibody-mediated rejection; aTCMR, acute T-cell mediated rejection; NR, non-rejection; yrs, years; aHUS, atypical hemolytic syndrome; MPO, Myeloperoxidase; IQR, interquartile range; HLA, human leukocyte antigen; CMV, Cytomegalovirus; SV40, simian virus 40; IFTA, Interstitial Fibrosis and Tubular Atrophy; n.a., not applicable; LsdL, Life screen de Luxe; LSA, Lifecodes Single Antigen; DSA, donor-specific antibodies; eGFR, Estimated Glomerular Filtration Rate. *According to Banff’19.

**Table 5:** Detailed immunological, histopathological and clinical information of individual patients included.

P.	group	LsdL class I (+/-/np)	LsdL class II (+/-/np)	DSA class I	MFI	DSA class II	MFI	DSA: p.e./dn	Days between biopsy/diagnosis and trans-plantation	Biopsy type	C4d	C3d glom	C3d ptc	C5b-9 glom	C5b-9 ptc
**1**	aABMR	+	+	B62	6000			dn	751	InB	C4d+	3	0	1	1
**2**	aABMR	+	+	A23	16000	DR53 DQ2	40000	dn	3095	InB	C4d+	3	1	1	0
**3**	aABMR	-	+			DQ2 DQ5	9000		440	InB	C4d+	2	0	1	1
**4**	aABMR	–	+			DQ2	3000	dn	1485	InB	C4d-	ms	0	1	0
**5**	aABMR	-	+			DQ6	10000	dn	3453	InB	C4d+	3	1	1	1
**6**	aABMR	+	+	B38, B51	40000	DR8 DR13 DR52	12000	dn	5	InB	C4d+	3	3	1	0
**7**	aABMR	-	+						5625	InB	C4d+	1	1	1	3
**8**	aABMR	+	–	B62	10000			dn	2256	InB	C4d+	3	1	1	0
**9**	aABMR	-	+	DQ1	20000			dn	3016	InB	C4d+	ms	ms	ms	ms
**10**	aABMR	+	–	A2	3000			dn	1006	InB	C4d+	3	1	1	0
**11**	aABMR	-	+			DQ6 DQ8 DR52	27900	dn	1499	InB	C4d+	3	1	1	1
**12**	aABMR	–	–						3297	InB	C4d+	ms	1	ms	1
**13**	aABMR	+	+			DR12 DR52 DQ9	14500	dn	7	InB	C4d+	3	1	ms	0
**14**	aABMR	+	+	A24,Cw9,Cw10	8000	DR13 DR52 DQ8	3500	p.e.	20	InB	C4d+	3	0	1	0
**15**	aABMR	+	+			DQ2	16000		969	InB	C4d+	1	1	1	1
**16**	aABMR	+	+	A24	2500	DR13 DR52	6000	p.e.	141	InB	C4d+	3	1	1	0
**17**	aABMR	+	+			DQ6	1000	dn	531	InB	C4d+	2	3	1	2
**18**	aTCMR	+	+						6	InB	C4d-	1	0	0	0
**19**	aTCMR	-	-						1921	InB	C4d+	1	0	ms	ms
**20**	aTCMR	–	–						154	InB	C4d-	1	0	0	0
**21**	aTCMR	-	-						883	InB	C4d-	1	0	1	0
**22**	aTCMR	–	–						305	InB	C4d-	ms	ms	ms	ms
**23**	aTCMR	+	+						451	InB	C4d-	1	1	ms	1
**24**	aTCMR	–	–						18	InB	C4d-	ms	ms	ms	ms
**25**	aTCMR	-	-						1111	InB	C4d-	3	0	1	0
**26**	aTCMR	–	–						15	InB	C4d-	1	0	1	1
**27**	aTCMR	-	-						77	InB	C4d-	1	1	0	0
**28**	aTCMR	–	–						55	InB	C4d-	0	1	0	2
**29**	aTCMR	-	-						142	InB	C4d-	3	1	0	0
**30**	aTCMR	+	+			DQ7	20000	dn	531	InB	C4d-	1	0	1	0
**31**	aTCMR	np	np						192	InB	C4d-	0	1	0	1
**32**	aTCMR	–	+			DR13	3000	p.e.	585	InB	C4d-	1	0	0	0
**33**	aTCMR	-	+			DQ2 DR53	4000	dn	117	InB	C4d-	2	1	0	1
**34**	aTCMR	–	–						11	InB	C4d-	2	0	1	0
**35**	aTCMR	-	-						18	InB	C4d-	0	0	ms	0
**36**	NR	np	np						387	PB	C4d-	1	1	1	0
**37**	NR	np	np						166	PB	C4d-	2	0	1	1
**38**	NR	np	np						357	PB	C4d-	0	0	1	0
**39**	NR	np	np						380	PB	C4d-	ms	ms	1	0
**40**	NR	–	–						372	PB	C4d-	0	0	0	0
**41**	NR	np	np						379	PB	C4d-	ms	ms	1	0
**42**	NR	np	np						392	PB	C4d-	1	0	1	0
**43**	NR	-	-						358	PB	C4d-	1	1	1	0
**44**	NR	–	–						388	PB	C4d-	1	0	0	0
**45**	NR	np	np						180	PB	C4d-	ms	0	ms	1
**46**	NR	np	np						169	PB	C4d-	1	0	1	1
**47**	NR	np	np						379	PB	C4d-	ms	ms	1	0
**48**	NR	np	np						193	PB	C4d-	ms	1	ms	0
**49**	NR	np	np						190	PB	C4d-	ms	ms	ms	ms
**50**	NR	np	np						178	PB	C4d-	1	0	ms	0

Histopathological diagnosis and Banff scores are based on Banff’19 classification. Anti-HLA-Abs are screened for in serum with LifeScreen de Luxe (LsdL). C4d deposition are displayed dichotomously in this table, indicating presence (+) or absence (-) in biopsy specimen. Complement factors C3d and C5b-9 were scored semiquantitatively. C3d and C5b-9 scores range from 0 to 4, indicating lack of any deposit, weak deposition (≤25%), moderate deposition (≤50%), substantial deposition (≤75%), and intense deposition (>75%) (25). p., patient; DSA, human-leucocyte antigen donor-specific antibody; MFI, mean fluorescence intensity; p.e., pre- existing; dn, deNovo; ABMR, Antibody-mediated rejection; aTCMR, acute T-cell mediated rejection; NR, non-rejection; IndB, biopsy due to clinical indication; protocol biopsy; +, positive test; -, negative test; np, test was not performed; ms, missing sample.

### Presence of HLA-Antibodies

LsdL-tests were performed on serum samples, which were taken at the same timepoints as plasma samples for aforementioned complement measurements. LsdL-tests for HLA-Abs class I and class II in serum were more commonly positive in aABMR patients when compared to TCMR and NR patients (*P<*0,01 respectively) (**Table 4**). Specifically, LsdL tests showed that 52,9% of ABMR patients were positive for HLA-Abs class I and 82,4% positive for HLA class II (**Table 4**).

HLA-Abs were identified as donor-specific in 70,6% of tested aABMR patients and in 16,7% of tested aTCMR patients. Respective mean MFIs of HLA class I of 13187,5 ± 12421 for aABMR and of HLA class II of 129991 ± 11711. For aTCMR with DSA class II, mean MFI was 90000± 9539. DSA-negative aABMR patients showed otherwise clear histopathological evidence for aABMR diagnosis according to the Banff’19 classification (**Tables 4**, **5**).

### Systemic Complement Activation

**Figure 2:**
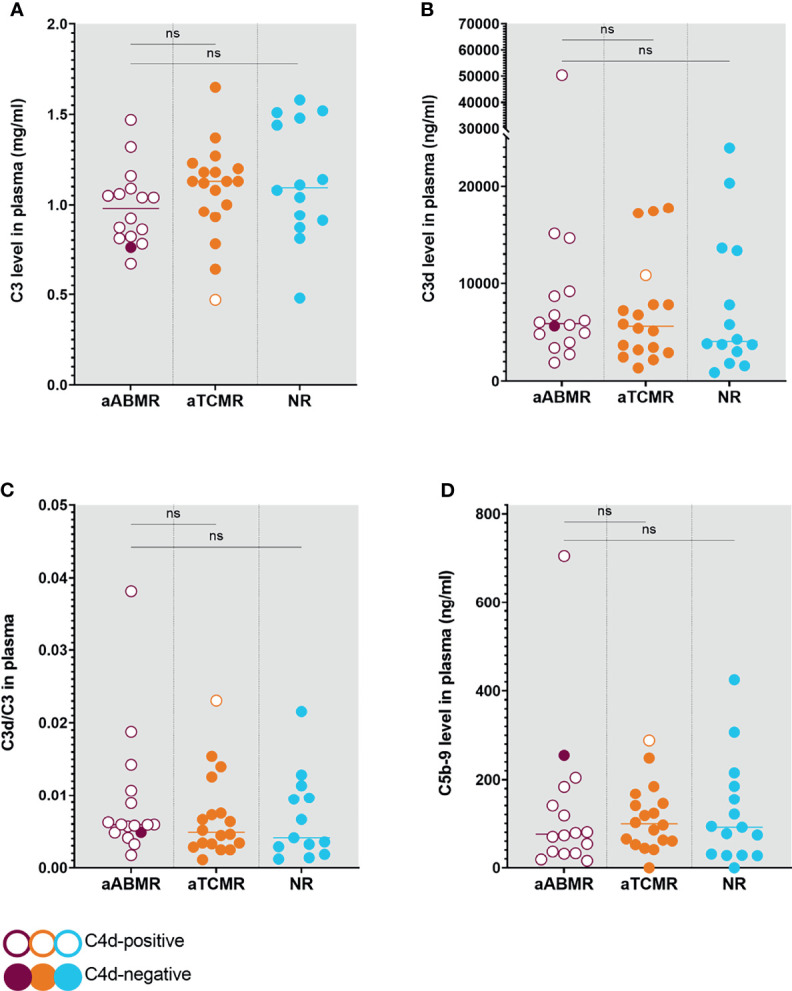
Complement Levels in Plasma of aABMR, aTCMR, and NR patients. Plasma levels were measured for C3 **(A)**, C3d **(B)**, and C5b-9 **(D)**. Ratios were calculated from respective C3 and C3d levels **(C)**, indicating systemic complement consumption. Circles represent individual patients with open circles indicating C4d-positive patients and filled circles symbolizing C4d-negative patients. P values depict results from Mann-Whitney U statistical analysis. Statistical significance is defined as P < 0.05. aABMR, active antibody-mediated Rejection; aTCMR, acute T-cell mediated rejection; NR, non-rejection; ns, not significant.

Quantification of plasma complement activation markers C3d, and C5b-9 did not show significant differences across groups. C3d/C3 ratio was evaluated as an indication for complement catabolism (26). Both C3 and C3d/C3 did not differ when comparing aABMR with aTCMR (*P*=0,7; *P*=0,4) and when comparing aABMR with NR (*P*=0,1; *P*=0,4) (**Figure 2**). Similarly, plasma C5b-9 did not differ when comparing ABMR with aTCMR (*P*=0,5) and with NR KTR (*P*=0,9). Plasma levels of C3d and C5b-9 complement factors, as well as C3d/C3 ratio, did not correlate with the intensity of complement deposition in renal biopsies of ABMR patients.

### Local Complement Deposition in Renal Biopsies

Biopsy samples were chosen with the closest temporal proximity to plasma samples, with a mean time difference of 3,9 ± 44 days. In a number of cases, complement deposition could not be determined due to missing biopsy material or due to missing glomeruli in available specimens (**Tables 3**, **5**).

C4d, as the standard clinicopathological marker, was scored according to Banff’19 (2, 15). C4d deposition was clearly linked to ABMR diagnosis, with 16 of 17 C4d-positive patients identified as aABMR patients (**Tables 3**, **5** **)**.

**Figure 3:**
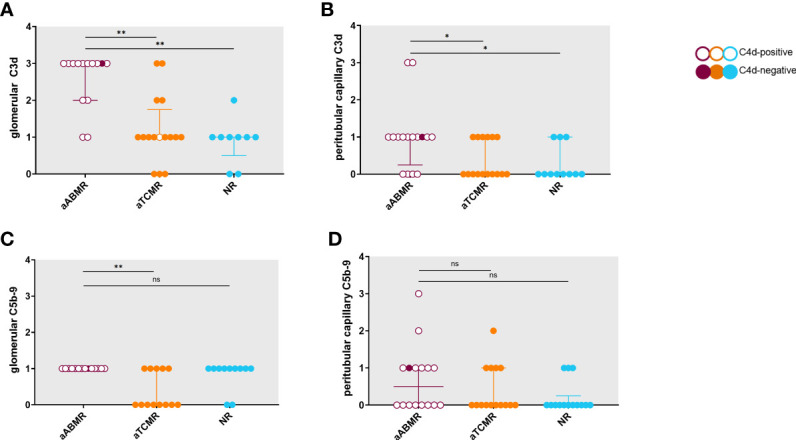
Semiquantitative scores for C3d and C5b-9 deposition in renal biopsy specimens of aABMR, aTCMR, and NR patients. Semiquantitative scores range from 0 to 4, with higher scores indicating increased positivity in biopsy. Groups are compared regarding glomerular C3d deposition **(A)**, peritubular capillary C3d **(B)**, glomerular C5b-9 **(C)**, and peritubular capillary C5b-9 **(D)** deposition. Data represent staining results of individual patients. Error bars represent median with interquartile range. Unfilled circles symbolize biopsies of C4d-positive patients, filled circles C4d-negative patients. P-values are derived from Mann-Whitney U tests with statistical significance defined as *P < 0.05, **P < 0.001; aABMR, active antibody-mediated rejection; aTCMR, acute T-cell mediated rejection; NR, non-rejection; ns, not significant.

The median score for glomerular C3d deposition in the aABMR group was 3, and in the aTCMR and NR group 1. Glomerular C3d deposition was significantly higher in the aABMR patients compared to aTCMR patients (*P<*0,01) and NR (*P<*0,01) (**Figure 3A**). The median score for C3d in the peritubular capillaries in the AMR group was 1, and in the aTCMR and NR group 0. Expression of C3d in peritubular capillaries was higher in aABMR compared to aTCMR (*P*=0,04) and NR patients (*P*=0,03) (**Figure 3B**). C3d expression appeared more intense in glomerular than in peritubular structures across all three groups (**Figure 3**).

Median scores for glomerular C5b-9 showed mild intensity (1) for aABMR and NR patients and negativity (0) for aTCMR patients. While glomerular C5b-9 deposition was not significantly increased in aABMR compared to NR patients (*P*=0,5), glomerular C5b-9 staining was stronger in aABMR compared to aTCMR patients (*P<*0,01) (**Figure 3C**). Peritubular capillary deposition of C5b-9 was similar across groups with median semiquantitative scores of 0 in all groups (**Figure 3D**).

**Figure 4:**
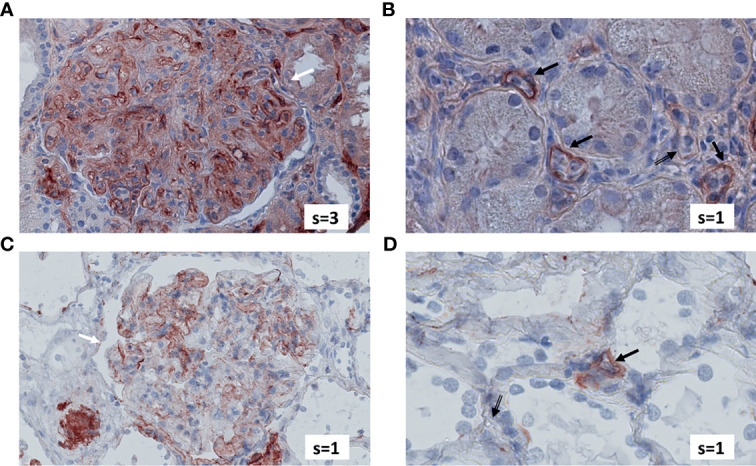
Renal biopsy staining for complement factors in a KTR diagnosed with C4d-positive, aABMR. Selected section of biopsy slide stained for C3d in glomeruli **(A)** and in peritubular capillaries **(B)**. Selected section of biopsy slide stained for C5b-9 in glomeruli **(C)** and in peritubular capillaries **(D)**. White arrows indicate glomeruli and black arrows indicate peritubular capillaries. Double-compound arrows indicate C5b-9 negative peritubular capillaries **(D)**. Semi-quantitative scores (0-4) are indicated in the lower right corner of each picture. KTR, kidney transplant recipients; aABMR, active antibody-mediated rejection; s, semi-quantitative score.

**Figure 5:**
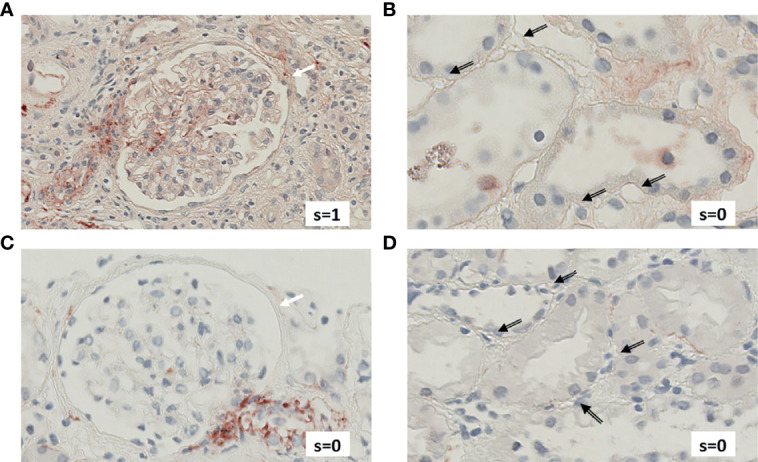
Renal biopsy staining for complement factors in a KTR diagnosed with aTCMR. Selected section of biopsy slide stained for C3d in glomeruli **(A)** and in peritubular capillaries **(B)**. Selected section of biopsy slide stained for C5b-9 in glomeruli **(C)** and in peritubular capillaries **(D)**. White arrows indicate glomeruli and black arrows indicate peritubular capillaries. Semi-quantitative scores (0-4) are indicated in the lower right corner of each picture. KTR, kidney transplant recipients; aTCMR, acute T-cell-mediated rejection; s, semi-quantitative score.

**Figure 6:**
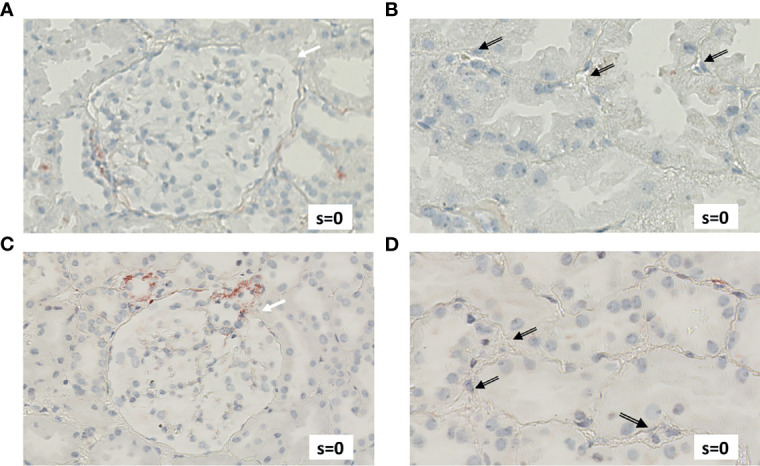
Renal biopsy staining for complement factors in a KTR without indication for undergoing biopsy and without signs of rejection in biopsy (NR patient). Selected section of biopsy slide stained for C3d in glomeruli **(A)** and in peritubular capillaries **(B)**. Selected section of biopsy slide stained for C5b-9 in glomeruli **(C)** and in peritubular capillaries **(D)**. White arrows indicate glomeruli and black arrows indicate peritubular capillaries. Semi-quantitative scores (0-4) are indicated in the lower right corner of each picture. KTR, kidney transplant recipients; s, semi-quantitative score.

**Figure 4** exemplifies the expression pattern in a C4d-positive aABMR patient, who presented with strong C3d deposition in the glomerulus, but only mild C3d in peritubular capillaries and scarce glomerular and no peritubular C5b-9. In comparison, in aTCMR and NR patients, C3d and C5b-9 were absent or only mildly expressed (**Figures 5**, **6**).

**Figure 7:**
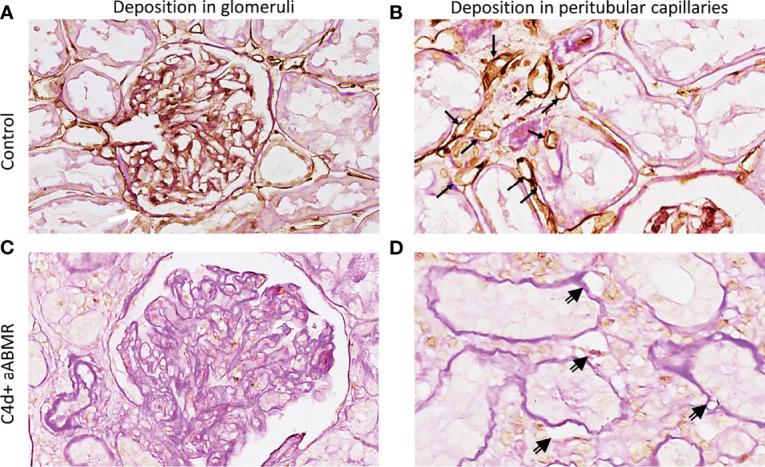
CD59 staining *in vivo*. Renal biopsy staining for complement regulator CD59 in glomeruli **(A, C)** and peritubular capillaries **(B, D)**. Staining was performed on human kidney before transplantation as control **(A, B)**, on biopsy specimen from a C4d-positive (C4d+) aABMR **(C, D)**. Black arrows point to CD59-positive peritubular capillaries, double-compound arrows to CD59-negative peritubular capillaries. aABMR, active antibody-mediated rejection.

Complement regulator CD59 in human renal tissue derived from a pre-transplantation biopsy is depicted in **Figures 7A, B**. CD59 was clearly expressed in both glomeruli and peritubular capillaries, with stronger deposition in peritubular structures (**Figure 7B**). CD59 staining in 5 aABMR patients showed a tendency of lower CD59 expression (**Figures 7C, D** **)** as compared to pre-transplantation control biopsy (**Figures 7A, B** **)**.

### Complement Deposition on CiGEnCs

**Figure 8:**
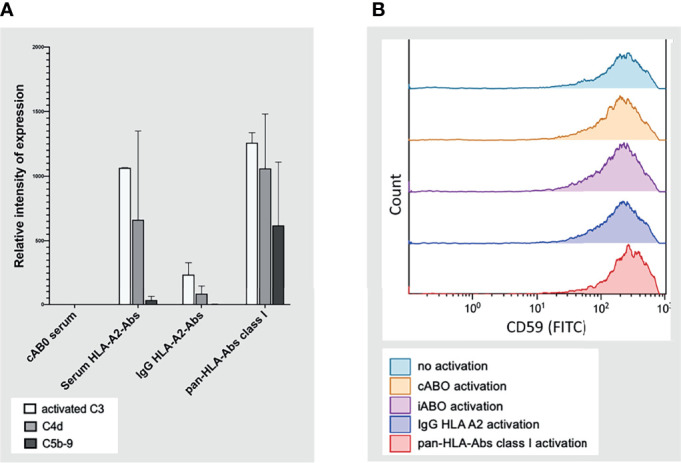
Complement system activation on conditionally immortalized glomerular endothelial cells *in vitro* in flow cytometric analysis. Complement factors C3 (activated), C4d, and C5b-9 on conditionally immortalized glomerular endothelial cells *in vitro* in flow cytometric analysis are depicted in **(A)** with the four different incubation conditions plotted on the y-axis. Deposition of complement regulator, CD59, was measured in flow-cytometry under five different incubation conditions **(B)**. cABO, ABO-compatible; HLA, Human Leucocyte Antigen; Abs, antibodies; IgG, Immunoglobulin G; FITC, Fluorescein isothiocyanate; iABO, ABO-incompatible.

Quantification of complement factors on CiGEnCs showed strong expression of C3d and C4d, but only slight or no deposition of C5b-9 after incubation with HLA-Abs. This expression pattern varied across the different incubation conditions but revealed a general trend of strong C3d and C4d with minor or absent C5b-9 expression. Notably, incubation with IgG HLA-A2 antibodies resulted in expression of complement factors C3d and C4d without simultaneous C5b-9 deposition on the cell surface. In the pan-HLA antibody incubation condition, C5b-9 expression was highest when compared to the other incubation conditions (**Figure 8A**). Complement regulator, CD59, was constitutively expressed on CiGEnCs, independent of the incubation condition **(** **Figure 8B** **)**.

## Discussion

In this study, we show robust proximal complement activation in aABMR renal biopsies compared to controls, without or only mild, concomitant C5b-9 deposition. The absence of co-expression of proximal and terminal complement factors might imply that terminal complement is less involved in aABMR pathogenesis. In this line of reasoning, the minor role of terminal complement could explain the disappointing efficacy of terminal complement inhibition as treatment for aABMR, particularly for late, aABMR (27–30).

Earliest research on immunohistopathology of ABMR by Feucht et al. revealed similar results. Next to their primary findings on the role of C4d deposition and its diagnostic value, Feucht et al. demonstrated the absence of C5b-9 deposits in C4d-positive ABMR patients (8).

Terminal complement regulation could account for the absence of C5b-9 deposits in aABMR patients, who did otherwise stain positive for C3d and C4d (**Figure 4**) (10, 31). Other authors indicated that complement regulation and degradation by CD59 could explain why C5b-9 deposition is volatile, discounting C5b-9 as a diagnostic marker in ABMR (10, 32, 33). Stronger protection against terminal complement by CD59 compared to the proximal regulators CD46 and CD55 might further explain why C3d and C4d but not C5b-9 was pronounced in aABMR (34).

We confirmed this assumed influence of CD59 *in vitro*, as it was continuously expressed on CiGEnCs, independent of the antibody stimulation condition. Similarly, we revealed clear endothelial CD59 expression *in vivo*, in pre-transplantation kidney biopsies (**Figures 7A, B** **)**. Interaction of CD59 and C5b-9 might cause internalization and/or degradation of the CD59/C9 complex, possibly explaining the absence of both C5b-9 and CD59 in ABMR biopsies. Particularly, Cai et al. showed that binding to the complement-binding side of CD59 can cause internalization of the complex in cancer cells (35). Despite these findings, literature on CD59 actions, degradation, and recycling processes is scarce. Thus, interpretations of CD59 expression in our aABMR samples remain speculative. Regarding the sustained CD59 positivity after complement activation *in vitro*, we assume that down-modulation by internalization of the CD59/C9 complex takes more time than the 45-minute time frame used in the *in vitro* complement activation experiments. In short, while the kidney endothelium seems to be highly protected against C5b-9 activation, this protection does not prevent aABMR. The absence of systemic and local C5b-9 fundamentally questions the relevance of terminal complement in aABMR pathogenesis (11, 31).

A broad body of evidence proved the efficacy of terminal complement inhibition in complement-mediated diseases like atypical hemolytic uremic syndrome or paroxysmal sleep hemoglobinuria (36, 37). In contrast, studies on the efficacy of terminal complement inhibition for ABMR presented with more equivocal results (27–30, 38, 39). Particularly, these studies were unable to show universal and enduring effectiveness of the terminal complement inhibitor, eculizumab (27–30). Interestingly, two case studies demonstrated substantial effectiveness of eculizumab in a pre-sensitized child (39) and a young adult (38), who showed clear deposition of immunoglobulins, C4d, and C5b-9 in renal biopsies. Complement deposits fully resolved within days after eculizumab administration in both case studies. Case studies in which eculizumab was effective as ABMR treatment, showed C5b-9 positivity, stressing the importance of C5b-9 involvement for eculizumab treatment (38, 39). Thus, we do not deny the potential relevance of identifying C5b-9-positive ABMR as an immunophenotypic subtype (40), although we showed that only a minority of our ABMR patients were C5b-9-positive.

Our *in vitro* results provide some indication for why C5b-9 deposition occurs in particular ABMR subgroups. Specifically, they suggest that a particularly high level of antibody-antigen interaction on the endothelial surface, as in the pan-HLA incubation condition, might be able to overwhelm any local complement regulatory barrier on endothelial cells. Overwhelming this regulation barrier possibly explains the mild expression of C5b-9 on endothelial cells after incubation with pan-HLA-Abs. Thus, C5b-9 might be involved in pathogenesis in specific ABMR patients, with early-onset rejection and/or with high titers of HLA-antibodies, in whom terminal complement inhibition might be relevant (41–43). In our cohort, a minority of aABMR patients presented with early acute rejection, partially because early acute ABMR is a rare event in recipients without pre-transplant HLA sensitization. Therefore, our findings are of particular relevance for the majority of ABMR cases in the clinical setting (41).

In C5b-9 negative aABMR, complement cascade split products like C4a, C3dg, iC3b, and C5a arising proximal to ultimate C5b-9 assembly might be sufficient to cause ABMR by binding to respective receptors on endothelium, creating a pro-inflammatory environment, or recruiting other immunogenic agents (33, 44–46).

Besides the complement system, alternative immunopathogenic pathways could be involved in aABMR, accounting for aABMR in the absence of C5b-9 in biopsies. A broad body of evidence emphasizes complement-independent mechanisms in ABMR pathogenesis, possibly mediated *via* Natural Killer cells (47–51). Others emphasized that DSA-binding could directly cause complement-independent endothelial cell activation (52–55).

To evaluate systemic evidence of complement activation in aABMR, complement factors in plasma were quantified, and no differences were found regarding C3, C3d, and C5b-9 levels between groups. This is in line with recently published findings revealing that ABMR and TCMR with circulating DSAs could not be distinguished by plasma complement factors (18)

Notably, aABMR patients showed increased expression of C3d in glomerular and peritubular structures compared to aTCMR and NR patients. C4d and C3d are considered stable split products that deposit on vascular structures after cleavage of C4 and C3 (11, 31). Pronounced C3d deposition in C4d-positive aABMR patients suggests that HLA-Abs cause classical pathway activation at least up to the level of C3 convertase in aABMR.

Other studies indicate that renal, C3d, and not, C4d deposition, could serve as a prognostic factor for graft functioning and survival in KTR with acute rejection (56, 57).

Our study has several strengths. First, we provide a sophisticated level of comparison for complement activation by including a well-defined, biopsy-proven cohort of aABMR, NR, and aTCMR patients. The aABMR group encompasses a relatively homogenous and clinically relevant group, with the majority of ABMR patients showing active or chronic active ABMR, and classical C4d- and DSA-positivity. Second, we provide a holistic picture of complement system activation, with factor quantification on local and systemic levels. This integrative complement analysis is crucial, considering that local and systemic complement system activation did not mirror each other. Third, we strengthened our findings *in vivo* by reproducing similar complement deposition patterns on glomerular, endothelial cells *in vitro*.

Some limitations of our study have to be noted. Our study was based on a small, single-center cohort, with some patients missing biopsy material. Future research should be performed on a larger group of ABMR patients to reflect and address the immunopathogenic heterogeneity of ABMR subgroups. Notably, we mainly included late, chronic active and active ABMR patients, whereas patients with chronic inactive ABMR, early ABMR and DSA-/C4d-negative ABMR are missing or underrepresented in our study. This underrepresentation limits any implications for these particular ABMR subgroups. Chronic active and active ABMR might differ in terms of the underlying complement-related pathogenesis, particularly as chronic active ABMR was more commonly C4d-negative (58). The possible influence of chronicity on complement activation cannot fully be addressed by our study due to the small sample size. However, as 16 of 17 included aABMR patients were C4d-positive, we do not expect that the chronicity component had a significant effect on complement analyses in our study. In addition, the Banff classification has its limitations and may not always represent the true underlying pathophysiology. For example, one patient classified as aTCMR on the basis of tubulitis and interstitial inflammation. This patient showed C4d-positivity in biopsy, but no other histological evidence for ABMR to fulfil the diagnostic criteria for aABMR. Within the boundaries of the Banff classification, the origin of C4d-deposition in this patient remains unexplained and a potential influence of undetected (non-)HLA-Abs cannot fully be excluded. With regard to the CD59 staining, it should be noted that it was only performed for 5 aABMR patients and preliminary results need to be validated in a larger aABMR population, as well as in aTCMR patients.

In conclusion, local C3d, and C4d are predominant hallmarks in the majority of aABMR patients, indicating upstream complement cascade activation of the classical pathway, which is not reflected by systemic complement activation. Terminal complement regulation, especially in peritubular capillaries, might explain this absence of C5b-9 despite C3d and C4d deposits. Thus, cytotoxicity is probably not mediated through C5b-9 in a significant proportion of aABMR patients. Together with our findings *in vitro*, our results provide sufficient justification to hypothesize that proximal complement factors or Fc-receptor mediated pathways could be more efficient pharmacological targets than terminal complement treatment.

## Data Availability Statement

The raw data supporting the conclusions of this article will be made available by the authors, without undue reservation.

## Ethics Statement

The studies involving human participants were reviewed and approved by University Medical Center Groningen (Groningen, The Netherlands) institutional review board (METc 2008/186). The patients/participants provided their written informed consent to participate in this study.

## Author Contributions

SB and JB were mainly responsible for the design of the study, as well as the supervision of the design of experiments, analyses, and writing processes. GT, RL, JK, AM-A, and FA carried out the experiments. MH conducted classifications and semi-quantitative scoring of biopsies. BH and RL were mainly responsible for quantification of HLA-antibodies in serum of patients. AD provided histopathological support for initial biopsy sample collection and selection of patients. RP provided advice for manuscript writing and the collection of pathological biopsy material. MD and MS provided advice in study design, analyses and writing of the manuscript. GT and RL were mainly responsible for writing, analysing, and designing figures and tables. All authors approved the final version of the manuscript.

## Funding

This study was supported by the Dutch Kidney foundation (grant number:13O CA27).

## Conflict of Interest

The authors declare that the research was conducted in the absence of any commercial or financial relationships that could be construed as a potential conflict of interest.

## Publisher’s Note

All claims expressed in this article are solely those of the authors and do not necessarily represent those of their affiliated organizations, or those of the publisher, the editors and the reviewers. Any product that may be evaluated in this article, or claim that may be made by its manufacturer, is not guaranteed or endorsed by the publisher.
