# Supplementary material for: Weak Expression of Terminal Complement in Active Antibody-Mediated Rejection of the Kidney
Source: Front Immunol. 2022 Apr 13;13:845301. doi: 10.3389/fimmu.2022.845301 (PMC9044906; doi:10.3389/fimmu.2022.845301)
Supplement: Supplementary file 1 [file DataSheet_1.docx]

Supplementary Material

# Weak Expression of the Terminal Complement in Active Antibody-Mediated Rejection of the Kidney

Gesa Tiller^1^, Rosa G.M. Lammerts^1,2^, Jessy J. Karijosemito^1^, Firas F. Alkaff^1,3^, Arjan Diepstra^4^, Robert A. Pol^5^, Anita H. Meter-Arkema^1^, Marc. A. Seelen^1^, Marius C. van den Heuvel^4^, Bouke G. Hepkema^2^, Mohamed R. Daha^6^, Jacob van den Born^1^, Stefan P. Berger^1^ on behalf of the COMBAT Consortium.

^1^ Division of Nephrology, Department of Internal Medicine, University Medical Center Groningen, Groningen, Netherlands

^2^ Department of Laboratory Medicine, University Medical Center Groningen, University of Groningen, Groningen, Netherlands

^3^Division of Pharmacology and Therapy, Department of Anatomy, Histology, and Pharmacology, Faculty of Medicine Universitas Airlangga, Surabaya, Indonesia

^4^Division of Pathology, Department of Pathology and Medical Biology, University of Groningen, University Medical Center Groningen, Groningen, Netherlands

^5^Department of Surgery, University Medical Center Groningen, University of Groningen, Groningen, Netherlands

^6^ Department of Nephrology, University of Leiden, Leiden, Netherlands

# Running title: Weak C5b-9 Expression in aABMR

# Manuscript: Weak Expression of Terminal Complement in Active Antibody-mediated Rejection of the Kidney

## Supplementary Material

# List of Contents:

## Supplementary Material 1. Banff-Scores of individual patients included according to the Banff’19 guidelines.

## Supplementary Material 2. In Vitro Study with Conditionally Immortalized Glomerular Endothelial Cells

## Supplementary Material 2.1. Cell Culture Handling

## Supplementary Material 2.2. Washing steps after HLA-incubation steps, as well as after incubation with FACS-labeling antibodies

## Supplementary Material 3. Positive and Negative Controls for the in-house C5b-9 staining Supplementary Figures and Tables

**Supplementary Material 1.** **Individual Banff scores for aABMR and aTCMR patients.** Individual scores were determined according to the Banff’19 guideline, evaluating glomerulosclerosis (gs), glomerulitis (g), mesangial matrix expansion (mm), glomerular basement membrane double contours (cg), interstitial inflammation (i), tubulitis (t), inflammation in the area of IFTA (i-IFTA), arteriolar hyalinosis (ah),intimal arteritis (v), vascular fibrous intimal thickening (cv), peritubular capillaritis (ptc), C4d and SV40. SV40, Simian virus; IFTA, interstitial fibrosis and tubular atrophy; n, number; a, arteries; TMA, thrombotic microangiopathy; aTCMR, acute T-cell mediated rejection; * biopsy slides of this aTCMR patients were not available for Banff-reclassification. ** no arteries present in the biopsy.

|  | Group | n(g) | gs | g | mm | cg | i | t | i-IFTA | ah | v | cv | ptc | C4d | SV40 | Histopathological diagnosis and Banff classification score |
| --- | --- | --- | --- | --- | --- | --- | --- | --- | --- | --- | --- | --- | --- | --- | --- | --- |
| 1 | aABMR | 12 | 1 | 0 | 0 | 0 | 1 | 1 | 3 | 3 | 0 | 1 | 2 | 3 | 0 | active ABMR |
| 2 | aABMR | 0 |  | 0 | 1 | 3 | 0 | 0 | 3 | 1 | 1 | 0 | 0 | 3 | 0 | chronic active ABMR |
| 3 | aABMR | 16 | 0 | 3 | 1 | 1 | 1 | 1 | 1 | 0 | 0 | 2 | 1 | 3 | 0 | active ABMR |
| 4 | aABMR | 15 | 2 | 1 | 1 | 2 | 0 | 0 | 0 | 2 | 0 | 1 | 2 | 0 | 0 | chronic active ABMR |
| 5 | aABMR | 13 | 2 | 0 | 1 | 3 | 0 | 0 | 1 | 1 | 0 | 0 | 0 | 3 | 0 | chronic active ABMR |
| 6 | aABMR | 9 | 0 | 0 | 0 | 0 | 0 | 0 | 0 | 0 | 0 | 0 | 0 | 3 | 0 | active ABMR |
| 7 | aABMR | 0 |  | 0 | 1 | 3 | 0 | 0 | 3 | 3 | 1 | 3 | 0 | 3 | 0 | chronic active ABMR |
| 8 | aABMR | 7 | 0 | 0 | 1 | 3 | 1 | 1 | 2 | 1 | 0 | 2 | 3 | 3 | 0 | chronic active ABMR |
| 9 | aABMR | 10 | 2 | 0 | 1 | 2 | 0 | 0 | 3 | 3 | 0 | 3 | 0 | 3 | 0 | chronic active ABMR |
| 10 | aABMR | 19 | 2 | 1 | 1 | 3 | 2 | 2 | 2 | 1 | 2 | 0 | 1 | 3 | 0 | chronic active ABMR, Banff IIB, IA |
| 11 | aABMR | 25 | 4 | 1 | 1 | 3 | 3 | 2 | 0 | 0 | 0 | 3 | 1 | 3 | 0 | active ABMR, Banff IA |
| 12 | aABMR | 0 |  |  |  |  | 0 | 0 | 0 | 0 | 1 | 0 | 1 | 3 | 0 | chronic active ABMR |
| 13 | aABMR | 9 | 0 | 0 | 0 | 0 | 1 | 0 | 0 | 0 | 1 | 0 | 0 | 3 | 0 | active ABMR, Banff IIA |
| 14 | aABMR | 12 | 0 | 0 | 0 | 0 | 1 | 0 | 0 | 0 | 0 | 0 | 0 | 3 | 0 | active ABMR |
| 15 | aABMR | 10 | 1 | 0 | 1 | 0 | 1 | 1 | 1 | 1 | 0 | 0 | 2 | 2 | 0 | active ABMR |
| 16 | aABMR | 7 | 0 | 0 | 1 | 3 | 0 | 0 | 0 | 0 | 0 | 0 | 0 | 3 | 0 | chronic active ABMR |
| 17 | aABMR | 6 | 0 | 0 | 1 | 0 | 1 | 1 | 1 | 1 |  |  | 0 | 3 | 0 | chronic active ABMR** |
| 18 | aTCMR | 8 | 1 | 0 | 0 | 0 | 1 | 1 | 0 | 0 | 1 | 0 | 1 | 0 | 0 | acute TCMR, Banff IIA |
| 19 | aTCMR | 8 | 0 | 0 | 0 | 0 | 3 | 3 | 1 | 0 | 0 | 0 | 0 | 3 | 0 | acute TCMR, Banff IB |
| 20 | aTCMR | 4 | 1 | 0 | 0 | 0 | 3 | 2 | 1 | 1 | 0 | 0 | 0 | 0 | 0 | acute TCMR, Banff IA |
| 21 | aTCMR | 12 | 0 | 0 | 0 | 0 | 2 | 3 | 0 | 0 | 0 | 1 | 0 | 0 | 0 | acute TCMR, Banff IB |
| 22 | aTCMR | 7 | 0 | 0 | 0 | 0 | 3 | 3 | 1 | 1 | 0 | 1 | 0 | 0 | 0 | acute TCMR, Banff IB |
| 23 | aTCMR | 10 | 1 | 0 | 1 | 0 | 2 | 2 | 2 | 1 | 0 | 3 | 0 | 0 | 0 | acute TCMR, Banff IA |
| 24 | aTCMR | 9 | 2 | 0 | 0 | 0 | 0 | 0 | 1 | 0 | 2 | 0 | 0 | 0 | 0 | acute TCMR, Banff IIB* |
| 25 | aTCMR | 16 | 1 | 0 | 0 | 0 | 3 | 3 | 1 | 0 | 0 | 1 | 0 | 0 | 0 | acute TCMR, Banff IB |
| 26 | aTCMR | 7 | 3 | 0 | 0 | 0 | 3 | 2 | 3 | 0 |  |  | 0 | 0 | 0 | acute TCMR, Banff IA** |
| 27 | aTCMR | 18 | 1 | 0 | 0 | 0 | 2 | 2 | 0 | 0 | 0 | 0 | 0 | 0 | 0 | acute TCMR, Banff IA |
| 28 | aTCMR | 5 | 0 | 0 | 0 | 0 | 1 | 2 | 1 | 0 | 1 | 1 | 1 | 0 | 0 | acute TCMR, Banff IA |
| 29 | aTCMR | 10 | 1 | 0 | 0 | 0 | 2 | 3 | 1 | 3 |  |  | 0 | 0 | 0 | acute TCMR, Banff IB** |
| 30 | aTCMR | 6 | 0 | 0 | 0 | 0 | 3 | 3 | 0 | 0 | 0 | 0 | 0 | 0 | 0 | acute TCMR, Banff IB |
| 31 | aTCMR | 5 | 1 | 0 | 0 | 0 | 3 | 2 | 0 | 0 | 0 | 3 | 0 | 0 | 0 | acute TCMR, Banff IA |
| 32 | aTCMR | 11 | 1 | 0 | 0 | 0 | 2 | 2 | 1 | 0 | 0 | 0 | 0 | 0 | 0 | acute TCMR, Banff IA |
| 33 | aTCMR | 11 | 0 | 0 | 0 | 0 | 3 | 3 | 0 | 0 | 0 | 1 | 0 | 0 | 0 | acute TCMR, Banff IB |
| 34 | aTCMR | 4 | 0 | 0 | 0 | 0 | 1 | 0 | 0 | 0 | 2 | 0 | 0 | 0 | 0 | acute TCMR, Banff IIB |
| 35 | aTCMR | 7 | 0 | 0 | 0 | 0 | 1 | 1 | 0 | 0 | 1 | 0 | 1 | 0 | 0 | acute TCMR, Banff IIA |

**Supplementary Material 2. In Vitro Study with Conditionally Immortalized Glomerular Endothelial Cells**

**Supplementary Material 2.1. Cell Culture Handling**

HLA and blood group typing of the CiGEnCs was determined using polymerase chain reaction (PCR) for sequence-specific oligonucleotide probes (SSOP) (Immunocor, Atlanta, USA) and polymerase chain reaction (6645, BAG health care, Lich, Germany), according to the manufacturer's protocol. Cells were cultured in a 6-wells-culture plate in EGM2- MV medium (EGM-2 endothelial med bullet kit, CC-3202, Lonza, 500 mL without VEGF supplemented) until they reached confluence and detached with cell dissociation solution (C5789, Sigma®, Zw jndrecht, The Netherlands) 900µl/1mL at 37°C. This cell suspension was collected in 4.5 mL tubes containing 2mL cell medium (EGM-2 endothelial med bullet kit, CC-3202, Lonza, 500 mL without VEGF supplemented) and centrifuged twice at 250 g for 6 min at 20°C.

**Supplementary Material 2.2. Washing steps after HLA-incubation steps, as well as after incubation with FACS-labelling antibodies**

Hereafter, cells were centrifuged twice for 6 min at 250 g at 20°C in culture medium, followed by incubation with 20% normal human serum as a complement source for 45 min at 37°C. After incubation, cells were washed once with 2 mL 20°C PBS/1% bovine serum albumin (BSA) (Fluorescence-automated cell sorting buffer) (Number, Sigma®, Zwijndrecht, The Netherlands) at 250 g for 6 min at 4°C and once with 2 mL 4°C FACS buffer at 250 g without a break for 6 min at 4°C. Before incubation with secondary labeling antibodies, two washing steps with ice-cold FACS buffer (Number, Sigma®, Zwijndrecht, The Netherlands) at 250 g without a break for 6 min at 4°C were performed.


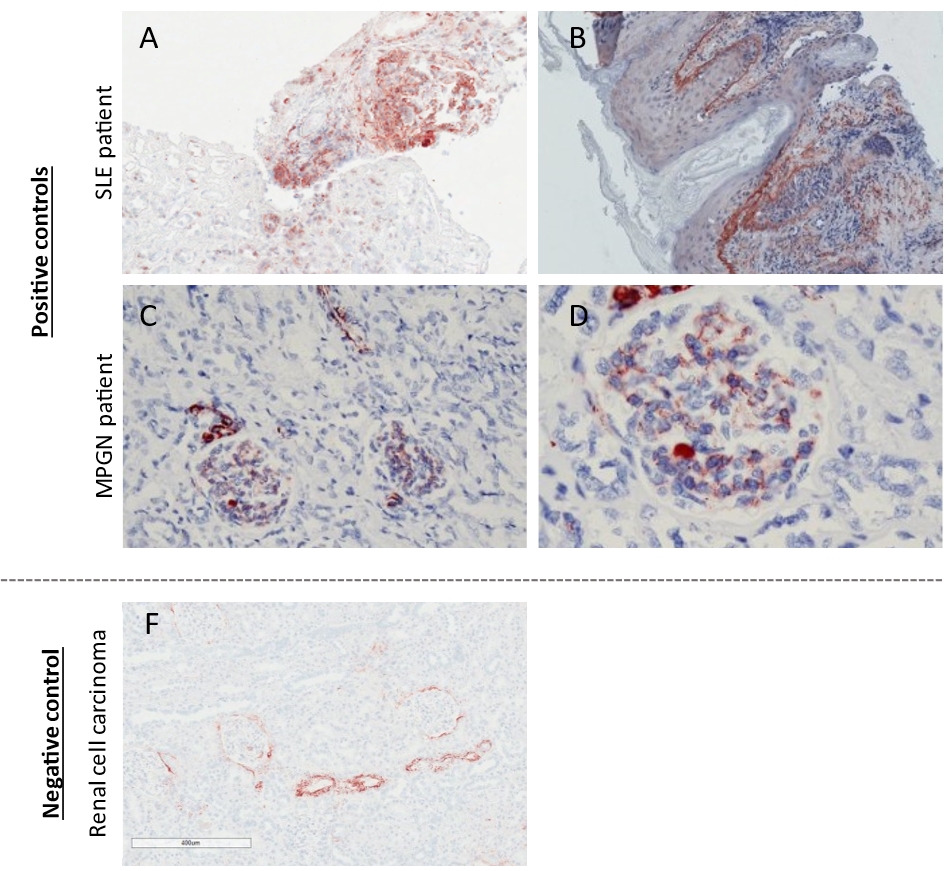


**Supplementary Material 3. Positive and Negative Controls for the in-house C5b-9 staining** Positive controls for the used C5b-9 in-house staining protocol as described in Table 2. The following tissues were used as positive controls with evident C5b-9 expression in A. renal biopsy tissue of a patient with diagnosed systemic lupus erythematosus, B. skin tissue of a patient with diagnosed systemic lupus erythematosus and C. renal biopsy tissue of a patient with membranous proliferative glomerulonephritis. As negative control for the in-house C5b/9 staining, renal cell carcinoma tissue was used and is depicted in 3.F. SLE, systemic lupus erythematosus; MPGN, mesangial proliferative glomerulonephritis.

**Supplemental Material 4. Time difference between date of blood and biopsy sampling for aABMR patients**. Date of blood sampling included the serological samples for anti-HLA-Abs diagnostics and plasma samples for complement measurements. Time difference is provided in days. n, number.

|  | time difference between blood and biopsy in days | | number of aABMR patients (n=17) | | Percentage (%) |
| --- | --- | --- | --- | --- | --- |
|  | | -7,00 | 1 | 5,9 | |
|  |  | -1,00 | 3 | 17,6 | |
|  |  | ,00 | 8 | 47,1 | |
|  |  | 1,00 | 3 | 17,6 | |
|  |  | 122,00 | 1 | 5,9 | |
|  | | missing | 1 | 5,9 | |
